# Elastic Band Exercises for Patients with Intensive Care Unit-Acquired Weakness: A Case Report

**Published:** 2018-02

**Authors:** Massimiliano Polastri, Stefano Oldani, Lara Pisani, Stefano Nava

**Affiliations:** 1 Medical Department of Continuity of Care and Disability, Physical Medicine and Rehabilitation, University Hospital St. Orsola-Malpighi, Bologna, Italy; 2 Department of Clinical, Integrated and Experimental Medicine (DIMES), Respiratory and Critical Care Unit, University Hospital St. Orsola-Malpighi, Bologna, Italy; 3 Alma Mater Studiorum University of Bologna, Bologna, Italy.

**Keywords:** Critical illness, Exercise movement techniques, Muscle weakness, Rehabilitation, Respiratory care units, Respiratory insufficiency

## Abstract

Intensive care unit-acquired weakness is characterised by severe impairment of muscle function that often arises after prolonged mechanical ventilation, difficult weaning, and severe sepsis. Elastic band exercises constitute an inexpensive and simple technique that is quite appealing for implementation in a “protected environment” such as the intensive care unit; however, elastic band application in the intensive care unit and in critical patients has not yet been described.

A 72-year-old male was referred to the respiratory intensive care unit for hypoxemic respiratory failure due to acute respiratory distress syndrome. Upper limb active exercises were performed using an elastic band exploring three main movement rays: abduction, forward flexion, and external rotation. At discharge, major improvements were observed for upper limb activities. The patient was also able to maintain a sitting position at the edge of the bed starting from day 27.

We found that an elastic band exercise program in a critical ill patient recovering from intensive care unit-acquired weakness was a suitable, safe, viable, and inexpensive therapeutic option to preserve residual upper limb motor activities and improve trunk control.

## INTRODUCTION

Intensive Care Unit-Acquired Weakness (ICUAW) is characterised by severe impairment of muscle function that often arises after prolonged mechanical ventilation, difficult weaning, and severe sepsis (1,2). Recent research has shown that propofol exposure may represent a risk factor for the onset of ICUAW in patients with sepsis (1). Critical illness myopathy and critical illness polyneuropathy syndromes characterise ICUAW, manifesting in flaccid generalised weakness and diminished tendon reflexes (1,2). It has been estimated that up to 1 million patients worldwide may develop ICUAW syndrome (2). Physical impairment caused by ICUAW can persist for an extended time (3). Patients who are exposed to a prolonged Intensive Care Unit (ICU) stay and/or develop ICUAW are more prone to fail to achieve a full recovery, with consequent reduced quality of life, which affects clinical, functional, and financial outcomes (4–7). Therefore, a rehabilitation program in an intensive care setting may be the cornerstone of ICUAW treatment (1,2). Elastic Band (EB) exercises have been described in many settings, including in therapeutic treatment of patients with compromised motor abilities (8–10). EB exercises generate activation of the prime mover, antagonist, stabiliser, and assistant mover muscles (11). EB exercises constitute an inexpensive and simple technique that is quite appealing for implementation in a “protected environment” such as the ICU; however, EB application in the ICU and in critical patients has not yet been described. In writing this case report, we followed the CARE criteria (12). The main objective of the current study was to evaluate the suitability of EB exercises in a patient with ICUAW.

## CASE SUMMARY

**Figure 1.**
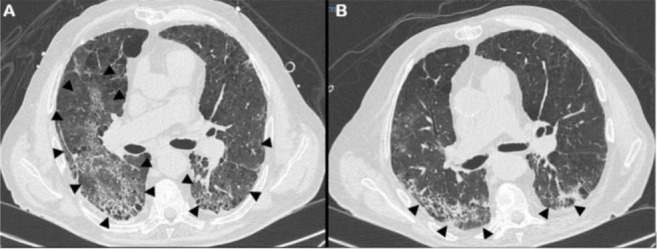
Pulmonary high resolution computed tomography. (A) Bilateral ground glass area affecting the inferior lobes (arrows). (B) Almost complete resolution of the ground glass area, and residual fibrotic phenomena (arrows).

**Figure 2.**
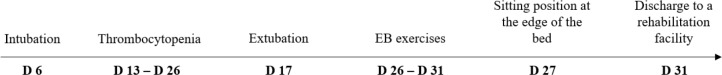
Timeline. D=day, EB=Elastic Band.

This was a case study performed at the University Hospital St. Orsola-Malpighi, Bologna, IT. The patient was informed about the study nature, and provided a signed informed consent. A 72-year-old Caucasian male with a body mass index of 27.9 (kg/m^2^) was referred to the Respiratory Intensive Care Unit (RICU) for hypoxemic respiratory failure due to acute respiratory distress syndrome. The past medical history included a heart transplantation 7 years earlier for arrhythmogenic cardiomyopathy, left atrial thrombus diagnosed 1 year earlier, arterial blood hypertension, a smoking habits of 15 packk/year, severe chronic obstructive pulmonary disease requiring long-term oxygen therapy, and a sleep breathing disorder treated with nocturnal mechanical ventilation. Medications on admission included warfarin, furosemide, and immunosuppressive therapy with tacrolimus, clomiphene, and prednisone. Physical examination showed respiratory distress with a respiratory rate of 34 breaths/minute and hypotension (arterial blood pressure 90/60 mmHg). An Arterial Blood Gas analysis (ABG), performed while the patient was breathing using a mask reservoir, revealed a partial pressure of oxygen (PaO_2_) reading of 112 mmHg, partial pressure of carbon dioxide (PaCO_2_) of 43 mmHg, pH 7.29, a partial pressure of oxygen/fraction of inspired oxygen (P/F) ratio of 112, and HCO_3_ of 19.9 mEq/L. High-Resolution Computed Tomography (HRCT) showed the onset of a bilateral ground-glass area affecting the inferior lung lobes (Figure 1A). Bedside echocardiogram revealed normal left ventricle function, with an ejection fraction of 61%, and a hypertrophic and hypokinetic right ventricle. Blood tests revealed a peak in serum phlogistic indices (C-reactive protein 13.7 mg/L, procalcitonin 13.9 ng/mL), along with thrombocytopenia (platelet count below 90,000/μL) and compromised renal function. After a brief trial with a noninvasive ventilation helmet, the patient developed sudden respiratory distress with confusion and agitation, and became haemodynamically instable and anuric. The patient was immediately intubated, and mechanical ventilation was performed (Figure 2). Dialysis was also required to treat acute kidney failure. The combined presentation of hypoxemia and bilateral infiltration, along with a history of immunodepression, was suggestive of a *Pneumocystis jirovecii* infection. Thus, trimethoprimsulfamethoxazole was initiated, and the prednisone dose was increased.

The clinical picture improved during the RICU stay; on day 17, the patient was extubated, and underwent the last dialysis on day 21 (Figure 2). Following extubation, muscular function deterioration was observed, resulting in severe motor impairment of the limbs (quadripareisis), absence of tendon reflexes, orbital fasciculations, and a significant increase in the myolysis indices (creatine phosphokinase 726 U/L; lactate dehydrogenase 449 U/L). Upper limbs muscle strength was evaluated as 2/5 referring the Medical Research Council scale (13); for the lower limb musculature there was a flaccid hypotonia of difficult assessment. The clinical frame was compatible with critical illness myopathy. The patient was at risk to develop skin ulcers, as the Braden Scale (14) score was 15 (cut-off score 16) on day 3.

**Figure 3.**
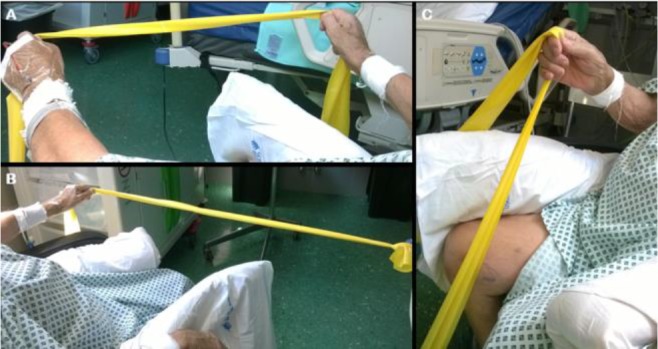
Elastic band exercises in a sitting position. (A) Arms opening. (B) Left shoulder external rotation. (C) Forward flexion.

A routine program of in-bed positioning had been planned over the first days to prevent complications related to prolonged bed rest, namely, joint and muscular retraction. Nursing staff was primarily involved in in-bed positioning. More complex rehabilitative procedures were avoided due to the patient’s haemodynamic instability, need for sedation, and risk of haemorrhage due to thrombocytopenia. Indeed, a large subset of patients during the first few days of mechanical ventilation may not undergo any exercise program due to respiratory or hemodynamic instability (15,16). Once the patient was extubated and cooperative, Range of Motion (ROM) exercises were performed to preserve residual motor activities and implement motor recovery. Due to the severe muscular impairment, the patient was not able to maintain a sitting position, and no postural transfers were possible at the time of extubation. Physiotherapy treatment was organised as follows: 1) passive/assisted ROM exercises (limbs) were performed from extubation until RICU discharge; 2) upper limb ROM exercises were performed using an elastic band (17). In this regard, a yellow band was considered suitable due to its 2.6 kg max resistance (if stretched by 250%). Supervised EB exercises were commenced on day 26 and continued until hospital discharge on day 31 (Figure 2). In the current case, it was possible to obtain an elastic band stretch of about 50–75%, resulting in resistance of approximately 0.8–1.1 kg (as reported by the manufacturer). For upper limb abduction exercises, it was possible to reach a maximum resistance of approximately 1.3 kg. Exercises were performed under continuous monitoring of the main vital parameters (heart rate, blood pressure, oxygen saturation), and it was interrupted in cases of severe fatigue or when the patient found it impossible to perform a movement. Each daily EB exercise session lasted about 30 minutes. Exercises were performed either in a supine or a sitting position to stimulate upper limb movements considering shoulder external rotation, forward flexion, and abduction (Figure 3). Abduction (movement out to the side of the body in a frontal plane) was the most challenging motor request for the patient, resulting indeed in a non-pure motion. EB exercises were performed every day using the same movement directions. A certain degree of progression was observed regarding ROM amplitude over the treatment, as described later in the text. Upper limb exercises were performed to improve upper body motor control and enhance postural transfers, allowing weight shift while in a sitting position. In addition, lower limb exercises were executed in a sitting position (patient was sitting in an armchair with a lifter) and consisted of ankle flexion–extension, knee raising, and leg extension; movements were always assisted, as it was not possible for the patient to actively complete the ROM. Considering this, it was not possible to use the EB for the training of the legs.

At RICU discharge on day 31, HRCT showed a decrease in bilateral ground-glass pulmonary lesions and improvements in lung ventilation (Figure 1B) which was obviously not related to the EB exercises. In addition, the Braden Scale score increased to 17; hence, the patient was no longer at risk of developing skin ulcers. On day 26, the platelet count increased above 150,000/μL, allowing for safe mobilisation and exercise. The subject could perform active in-bed postural variations to prevent worsening of the Braden score. The patient was also able to maintain a sitting position at the edge of the bed starting from day 27 (Figure 2).

At RICU discharge, the right arm had approximately 30 degrees of abduction, and the left-side ROM was greater, reaching 90 degrees. On the right side, the maximal elastic band lengthening was 117% for abduction, producing approximately 1.3/1.5 kg resistance during movement execution; it was 40% (0.5/0.8 kg) for forward flexion and external rotation. On the left side, the forward flexion produced an elastic lengthening of 25% (0.5 kg), while external rotation performance was 30% (0.5 kg). Variations in band lengthening were calculated using a tape meter by measuring the elastic band length at rest and at the maximal elongation during exercise: metric differences in length are reported as a percentage. Nevertheless, when the patient was discharged to a rehabilitation facility on day 31 to continue the physiotherapy treatment, he was not able to autonomously perform complex postural variations. No adverse events were observed related to the EB exercise sessions, and the treatment was well tolerated by the patient, who adhered to it with full cooperation.

## DISCUSSION

To the best of our knowledge, there are no previous studies exploring the feasibility of an EB exercise program in an acute setting in patients with ICUAW. Rehabilitation was shown to improve functional independence at discharge, increase ventilator-free days, and increase walking distance (18). However, this was possible in a very experienced unit with a large and dedicated multidisciplinary staff (19); therefore, these results may not be achievable in environments where the human and financial resources are limited. This case report demonstrates that rehabilitation can also be achieved in patients with ICUAW using a “low cost” device and without a dedicated team. The main advantages of using the EB exercise were the active involvement of the patient and the capacity to measure the workload during exercise due to the mechanical properties of the elastic band. In addition, it was possible to calculate in kg the resistance offered by the EB for each movement direction. During EB exercises, muscle tension varies according to the band length throughout the ROM (11). We found that EB exercises were an appropriate approach to achieve initial motor recovery in the current case. It should be highlighted that elastic resistance can be adapted to the patient’s characteristics, providing adequate initial tensioning, establishing a defined lengthening goal, or using different bands with different resistance properties. The bell-shaped curve of muscular contraction strength that can emerge during EB exercises (11) was useful in our case to stimulate muscle activation in the non-compromised degrees of ROM. A bell-shaped strength curve represents a mechanical condition where the movement is easier at the middle phase of the execution, while the end and beginning are more difficult, as muscle contraction varies in relation to tension. In this case, EB exercises allowed for safe training and avoided inappropriate effort. Another advantage of using EBs is the opportunity to obtain continuous motor control by asking the subject to maintain tension in the band, even in cases where the ROM is severely impaired. Reinforcement and neuromuscular activation of the upper limbs is required to maintain a sitting position and to allow for a continuing, and more intensive, rehabilitation program. In the present case, EB exercises were executed using a yellow band, which at minimum lengthening (25%) could provide resistance of 0.5 kg. Over time, we would surmise the patient will be able to execute more intense elastic training using more-resistive bands, such as the green and/or blue bands, which generate minimum resistance of 0.9 and 1.3 kg, respectively. Although the EB exercises have been carried out with regularity, in the current case it was not possible to standardize a specific protocol with defined characteristics regarding number of repetitions, items, and duration: this limits the reproducibility of the program illustrated here. Nevertheless, we tried to standardize some aspects: for example, the starting position for the movements execution within the three main directions (abduction, forward flexion, and external rotation) was always in sitting with the upper limbs along the sides. Furthermore, a head of the EB was hold by the physiotherapist or by the patient itself (when possible); the patient was asked to perform the movement in a certain direction trying to reach the maximum ROM, using the other elastic head. During the exercise execution, the patient was instructed and supported verbally to reinforce the technical aspects of the movements. In one case the patient was able to form a grip, hold both ends and perform bilateral upper limbs exercises (Figure 3A). It could be surmised that exercise effect will be different when obtained using a bilateral versus a unilateral movement: in the current case, we have encouraged both modalities to maximize the muscular reinforcement, as well as trunk proprioception. Exercise precautions and progression were discussed daily within a Multidisciplinary Team (MDT) composed of a physiotherapist, intensive care physician, and nursing staff. Therapeutic goals were discussed within the MDT, planning different activity intensities according to the patient’s daily clinical picture. Continuous monitoring and scheduling of a step-wise and individualised recovery pathway were crucial to achieving optimal results, as previously described. In addition, the team culture played an important role in the appropriateness of care, as team members shared professional competencies.

The present report has several limitations, the main concern being the lack of consistent outcome measures. However, assessing and quantifying physical performance in critically ill patients is challenging, as confirmed by our results. To provide a reliable assessment of the patient’s physical condition and physical performance, we evaluated the ability to maintain a sitting position independently at the edge of the bed as an outcome using a qualitative evaluation (yes/no); this was possible before discharge. Another limitation is the absence of a long-term follow-up; in this regard, we were forced to observe only the first few days after EB exercises were commenced because the patient was discharged to a rehabilitation facility to complete the physiotherapeutic program. Furthermore, one more ‘technical’ limitation is present in this case report, as the figures illustrated here are conveying a static point of the exercises; on the other hand, due to the patient’s condition we were not able to make a more detailed and animated representation of the exercises execution. Indeed, pictures have been taken with the purpose to exemplify the directions of the movements: to this end, it should be highlighted that some directions illustrated in the Figure 3 are not perfectly corresponding to the intended movement (*i.e.*, abduction). Finally, due to the absence of previous published studies on the topic, we cannot compare our findings with other studies, nor can we extend our assumptions to a wider population, as they are based on a single case study.

## CONCLUSION

In the present case report, we have illustrated the execution of EB exercises for a critical patient in a protected environment such as the RICU. Despite the limitations of the current case study, we found that an EB exercise program in a critical ill patient recovering from ICUAW was a suitable, safe, viable, and inexpensive therapeutic option during the RICU stay to preserve residual upper limb motor activities and improve trunk control. Further experimental studies can increase our understanding on the effectiveness of EB exercises in critical ill adult patients with ICUAW, which has not yet been explored.
